# Health Policy for Prostate Cancer Early Detection in the European Union and the Impact of Opportunistic Screening: PRAISE-U Consortium

**DOI:** 10.3390/jpm14010084

**Published:** 2024-01-11

**Authors:** Katharina Beyer, Renée Leenen, Lionne D. F. Venderbos, Jozien Helleman, Frederique Denijs, Wichor Bramer, Vera Vasilyeva, Erik Briers, Juan Gomez Rivas, Renata Chloupkova, Ondrej Majek, Lieven Annemans, Pieter Vynckier, Partha Basu, Arunah Chandran, Roderick van den Bergh, Sarah Collen, Hendrik van Poppel, Monique J. Roobol, on behalf of the PRAISE-U Consortium

**Affiliations:** 1 Department of Urology, Erasmus MC Cancer Institute, University Medical Center Rotterdam, 3015 GD Rotterdam, The Netherlands; r.leenen@erasmusmc.nl (R.L.); l.venderbos@erasmusmc.nl (L.D.F.V.); j.helleman@erasmusmc.nl (J.H.); f.denijs@erasmusmc.nl (F.D.); roodvdb@hotmail.com (R.v.d.B.); m.roobol@erasmusmc.nl (M.J.R.); 2 Medical Library, Erasmus MC, University Medical Center Rotterdam, 3015 GD Rotterdam, The Netherlands; w.bramer@erasmusmc.nl; 3 European Association of Urology, Policy Office, 6842 CV Arnhem, The Netherlands; v.vasilyeva@uroweb.org (V.V.); s.collen@uroweb.org (S.C.); hendrik.vanpoppel@kuleuven.be (H.v.P.); 4 Europa Uomo, 2018 Antwerp, Belgium; erikbriers@telenet.be; 5 Department of Urology, Clínico San Carlos University Hospital, 28040 Madrid, Spain; juangomezr@gmail.com; 6 National Screening Centre, Institute of Health Information and Statistics of the Czech Republic, 128 01 Prague, Czech Republic; renata.chloupkova@uzis.cz (R.C.); ondrej.majek@uzis.cz (O.M.); 7 Institute of Biostatistics and Analyses, Faculty of Medicine, Masaryk University, 625 00 Brno, Czech Republic; 8 Department of Urology, Gasthuisberg University Hospital, Katholieke Universiteit Leuven, 3000 Leuven, Belgium; lieven.annemans@ugent.be (L.A.); pieter.vynckier@ugent.be (P.V.); 9 International Agency for Research on Cancer, World Health Organization, 69366 Lyon, France; basup@iarc.who.int (P.B.); chandrana@iarc.who.int (A.C.)

**Keywords:** prostate cancer, screening, early detection, policy, health policy

## Abstract

With the new policy recommendation in 2022 to explore the possibilities of screening for prostate cancer by the European Commission, the landscape for prostate cancer early detection is evolving. In line with this recommendation, the PRAISE-U project aims to evaluate the early detection and diagnosis of prostate cancer through customised and risk-based screening programmes, with the goal to align protocols across European Union member states. This systematic review is part of the PRAISE-U project, with the goal to review the policy, medical guideline recommendations, and the current level of opportunistic screening presented in the scientific literature on prostate cancer early detection from 2016 to 2023 in European Union member states. An extensive literature search was performed on 1 June 2023 in a large number of databases, including Embase.com, Medline (Ovid), Web of Science Core Collection, Google Scholar, and Policy Commons. We identified 318 articles (qualitative, quantitative, and reviews), of which 41 were included in the full-text screening. Seventeen articles were ultimately identified as eligible for inclusion. The included articles revealed significant variations towards PSA-based early detection policies for prostate cancer in nine European countries. Despite official recommendations, opportunistic screening was prevalent across all nine countries regardless of recommendations for or against PSA-based early detection. This systematic review suggests that the current early detection policies are not fit for purpose. High levels of opportunistic screening and overdiagnosis persist, prompting policy recommendations for standardised guidelines, informed decision making, and increased awareness to improve efficiency and effectiveness in early detection.

## 1. Introduction

In 2020, prostate cancer ranked as the second most common cancer globally and the fifth leading cause of cancer-related deaths among men [1]. International differences in prostate cancer diagnostic practices likely contribute to the variation in prostate cancer incidence rates [2]. The landscape of prostate cancer diagnosis underwent a transformative shift in the late 1980s and early 1990s with the introduction of the prostate-specific antigen (PSA) test [3,4,5,6,7]. This breakthrough enhanced the early detection of PCa, allowing for timely intervention and treatment [7]. However, it has also sparked ongoing debates and concerns regarding the issue of overdiagnosis and overtreatment, which continues to be a significant discussion in prostate cancer management [7,8]. This debate has been heavily influenced by two randomised controlled trials (RCTs) with the same primary end point ‘prostate cancer specific mortality’: the European Randomised Study of Screening for Prostate Cancer (ERSPC) in Europe and the US-based Prostate, Lung, Colorectal, and Ovarian (PLCO) Cancer Screening Trial in the United States (US) [9].

ERSPC has consistently demonstrated a significant relative risk reduction of 20% in PCa-specific mortality in favour of screening. This benefit persisted after a 16-year follow-up. Moreover, the trial revealed a 30% relative reduction rate in metastatic prostate cancer (M+) [10]. In contrast, after the first 10 years, the results published by the PLCO did not show a substantial difference between the study group (undergoing annual screening) and the control group (receiving usual care). This led the United States Preventive Services Task Force ((USPSTF) to recommend against PSA screening in 2012. However, a significant criticism directed at this trial centred on the extent of PSA testing in the control group. Subsequent analyses in 2016 revealed that “approximately 50% of men in the control group received at least one PSA test during the study” [11], prompting a re-analysis of the results and a correction of the conclusions. Ultimately, both trials showed a reduction in mortality of similar magnitude [9].

This debate was also reflected upon at the European Union policy level; the new evidence in 2016 sparked the need to revisit policy recommendations, and the European Council requested the Scientific Advice for Policy by European Academies (SAPEA) to conduct a comprehensive review of the current scientific evidence regarding prostate cancer screening [9,12].

**Figure 1:**
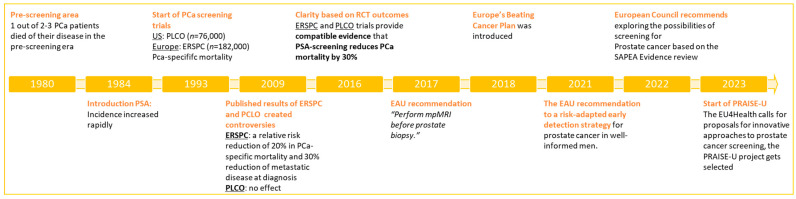
Overview of the history of PSA early detection.

SAPEA’s review served as the foundation for the updated recommendation by the Council of the European Union (2022) to recommend member states to evaluate the feasibility and effectiveness of organised prostate cancer screening [12]. In line with this recommendation, the European Commission opened a call to further investigate screening in PCa. The Prostate cancer Awareness and Initiative for Screening in the European Union (PRAISE-U project) was selected to evaluate the early detection and diagnosis of prostate cancer through customised and risk-based screening programmes within the framework of organised programmes, deescalating the ongoing unregulated and opportunistic screening (www.uroweb.org/praise-u, accessed on 27 November 2023) (see Figure 1) [9,12,13].

This systematic review is part of the PRAISE-U project, with the aim of reviewing the policy, medical guideline recommendations, and the current level of opportunistic screening published in the scientific literature on prostate cancer screening from 2016 to 2023 in European Union member states.

## 2. Methods

This systematic review was reported in accordance with the PRISMA guidelines and has been published under PROSPERO CRD42023440555.

### 2.1. Search Strategy

An extensive literature search was performed on 1 June 2023, using Embase.com, Medline (Ovid), Web of Science Core Collection, Google Scholar, and Policy Commons. The cut-off for the inclusion of manuscripts was 2016. This date was chosen as this was when the new evidence from ERSPC and PLCO aligned. The main structure of the search strategy comprised concepts such as: (1) PCa; AND (2) Early Detection OR Screening; AND (3) Policy OR Politics, AND (4) the 27 European Union member states (see Supplementary Materials S1 for the detailed search strategy). The supplementary search approach included reference list checking and contacting experts.

To design the search strategy and identify studies, we worked with an information scientist from the Erasmus MC Medical library (WB), who also removed duplicates using the method by Bramer et al. in EndNote [14].

### 2.2. Selection Criteria

This systematic review focused on quantitative and qualitative studies published after January 2016 that were conducted within the 27 member states of the European Union. The study design encompassed both quantitative and qualitative studies as well as reviews to comprehensively explore this topic (see Table 1).

The final list of identified studies was assessed independently by two researchers (KB and RV) for abstract and full-text screening. Disagreements were resolved by discussion, and if a consensus could not be reached, a third reviewer (LV) was consulted.

### 2.3. Data Extraction

Data from included studies were extracted by one member of the research team (KB) using a standardised data extraction form, including study design, setting, subjects, policy recommendations, interventions, outcomes measured, and results, including contextual factors, and these were reviewed by a second reviewer (RL). Themes were identified, and the data were narratively described. Any disagreements were again resolved by consulting a third reviewer (LV). Due to the nature of the review and the information retrieved, i.e., information on policies and the state of play containing terminology, we did not perform a Risk of Bias assessment, as no estimation of the effect size of treatments was conducted.

## 3. Results

**Figure 2:**
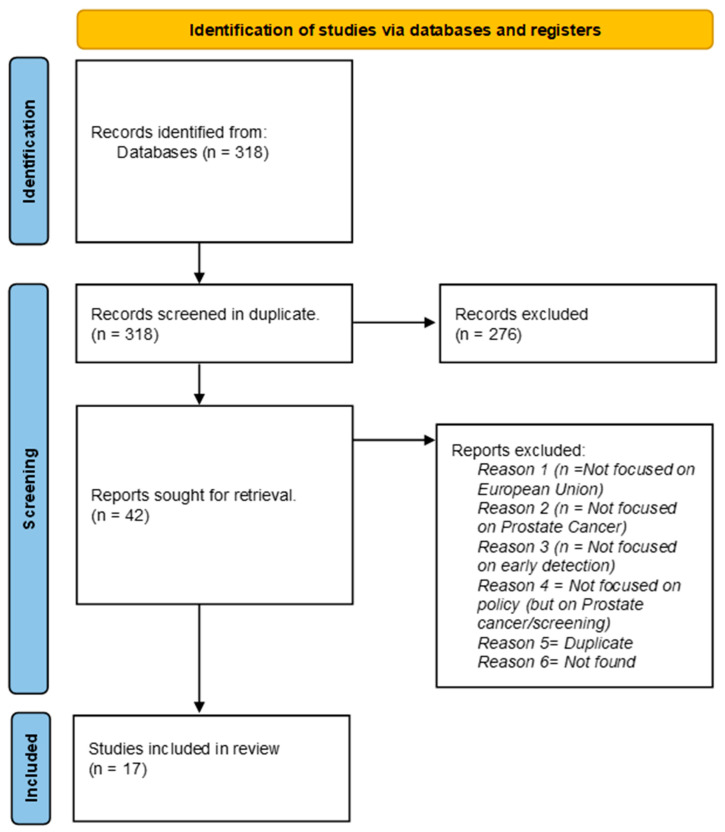
PRISMA diagram.

We identified 318 articles, of which 41 were included in full-text screening. Seventeen articles were ultimately identified as eligible for inclusion (see Figure 2). These articles reported on the screening policies for prostate cancer and/or the current state of prostate cancer screening in a given country or across the European Union. Out of the seventeen articles, one reported the policies and the state of play looking at the European Union level, three explain the situation in Germany, two in France, two in the Netherlands, one comparing the approaches in the Netherlands and Germany, and one each in the following countries: Croatia, the Czech Republic, Germany, Italy, Ireland, Lithuania, Portugal, and Spain.

For analysis, we present a narrative summary that discusses the patterns observed in the data.

Variations in PSA-based screening policies (individual early detection or population strategies).

**Figure 3:**
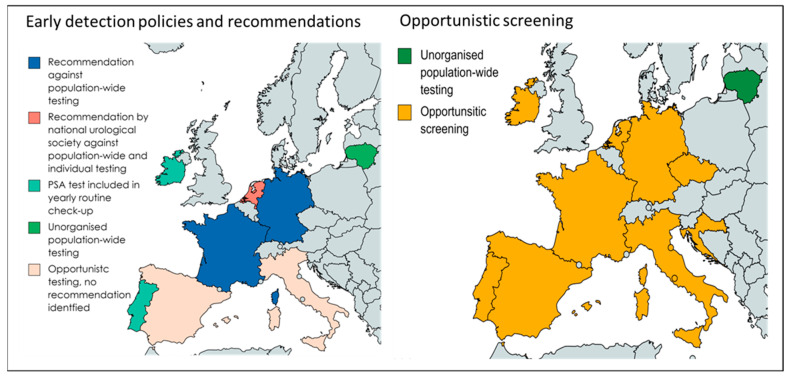
Overview of early detection.

The included literature highlights significant variations in how European countries approach the early detection of prostate cancer using PSA tests (see Figure 3). Moreover, a review by Albreht T. et al. [15] in 2021 emphasised that, overall, prostate cancer has not received as much attention in European cancer control plans as it should, despite its significance in terms of mortality, incidence, impact on quality of life, and healthcare costs [15].

We identified three European Union countries where studies have highlighted respective policies and national guidelines (Germany, France, and the Netherlands) [16,17,18]. Other countries either do not have a clear recommendation or such recommendations have not been communicated clearly in the literature included.

Germany and France have clear recommendations against population-based PSA screening from their respective healthcare authorities. The German Institute for Quality and Efficiency in Health Care (IQWiG) and the French Haute Autorité de Santé (HAS) have discouraged PSA screening since 2020 and 2013, respectively [16,17,18]. Both authorities have reached the consensus that prostate cancer screening using a PSA test causes significant harm through overdiagnosis, outweighing the benefits of an earlier cancer diagnosis. This is also reflected in the medical guidelines from these countries, which recommend against systematic screening.

The French Committee of Urologic Oncology has in the meantime revised its recommendation based on individual early testing. It now advocates that PSA testing should be considered after providing individuals with detailed information about the potential benefits and harms of the test. Furthermore, individual risk factors should be taken into account when deciding whether to proceed with a PSA test or not [18].

Similarly, the German Urological Society guidelines (S-3 Leitlinien) recommend urologists to proactively inform men of PSA testing as an individual screening method, whereas, in the same guideline, primary care physicians are informed to not proactively raise this issue with their patients unless they inquire about screening [19].

In the Netherlands, the Dutch Urological Association guidelines (Nederlandse Vereniging voor Urologie) recommend against actively offering PSA testing to men without clinical symptoms of PCa, which suggests, according to Kappen et al. [20], that the Dutch guidelines are stricter in their approach to PSA screening [20].

In Ireland, the Irish Cancer Society suggests informed decision making and an active discussion on an individual level with men from the age of 40 to 50 depending on risk factors. However, routine testing is not advised for men older than 70–75 years [21].

### 3.1. What Is Happening in Practice?

PSA testing is actively used and often also increases over time across all countries reported in the included studies. According to the literature, it seems that across the European Union, the awareness of PSA testing is high among men, and many are willing to undergo testing. Factors influencing willingness to undergo the test include perceived usefulness of the test, personal health status, and desire for more information [15].

In the Netherlands, the authors report that the overall PSA testing rate in men aged ≥45 years has increased significantly from 2002 to 2011, despite recommendations for more conservative use of PSA testing [20].

Germany has a significant level of opportunistic PSA screening, even including men aged over 75 years, although evidence suggests that this age group is least likely to benefit [19]. A study in the German Münster district conducted by Simbrick et al. [22] revealed that 30.6% of men aged 45 years and older had a PSA value determined within the last 12 months (data from 2013) [22]. Over half of the PSA determinations that could be attributed to opportunistic screening occurred outside the recommended age group of 55–69 years, which is considered the target group for effective PSA screening according to the ERSPC. The data also showed that two-thirds of cancer screening examinations were conducted in general practitioner (GP) practices, with only about one-third occurring in urological practices [22,23].

Similar to Germany, France does not have a national prostate cancer screening program, but opportunistic PSA testing is widely practised. According to Tuppin et al. [17] (2017), in 2014, approximately 27% of the 11.6 million men aged 40 years and older in France underwent at least one total PSA test, and 5.6% underwent at least one free PSA test [17]. The rates of testing varied significantly depending on the presence or absence of treated lower urinary tract symptoms (LUTSs), with higher rates in individuals with LUTSs (53% for total PSA and 15% for free PSA) compared to those without (24% for total PSA and 5% for free PSA) [18].

GPs were responsible for advising 91% of the PSA tests reimbursed in 2014 (92% for total PSA and 87% for free PSA), while urologists ordered only 4% of the reimbursed tests [17].

In Croatia, PSA testing was widely introduced in 1990, and there seems to be a dogmatic practice of regular annual PSA testing for men over 50, regardless of recent PSA values, leading to a high volume of opportunistic screening. However, this approach of unorganised testing has not led to the expected improvement in the incidence, mortality, prevalence, and survival of prostate cancer, as reported by Reljić et al. [24].

Morlando et al. [25] surveyed men in Naples, Italy, to assess the knowledge, attitudes, and practices of PSA testing among men. They found that 72.7% of the respondents were aware of the PSA test, with 51.1% learning about it from their physicians. However, only 29.6% of the men had undergone a PSA test, while 59.4% expressed willingness to do so in the future [25].

In Portugal, routine blood tests are conducted annually and include PSA screening [26]. Interestingly, Conde et al. [26] observed a correlation between the request for routine blood tests and the request for various laboratory tests intended for screening purposes, such as PSA. This association suggests that physicians who order routine blood tests may also be intending to screen for various pathologies, even in asymptomatic patients without apparent risk factors. They also identified that most doctors who do not prescribe routine laboratory tests do not support screening for asymptomatic individuals for PCa, which aligns with existing evidence [26].

In Ireland, Connolly et al. [21] reported that there is no national prostate cancer screening program. However, 71% of older men received a PSA test or DRE from their GP between 2017 and 2019, and some, depending on their health insurance (i.e., publicly insured), would have to pay for such a GP visit [21].

In 2006, Lithuania initiated an organised nationwide PSA-based Early Prostate Cancer Detection Programme (EPCDP) operating on an opportunistic approach, targeting its 2.8 million inhabitants in 2019. The screening for prostate cancer was made available by general practitioners during regular visits for any medical concerns. Over a span of 10 years, approximately 70% of men aged 50–74 years participated in the screening at least once. Among the tested individuals, less than 17% showed a positive PSA test result, leading to diagnoses of prostate cancer in 9–13% of those cases [27].

A multicentre study conducted in seven areas of Castilla–León, Spain, identified a high incidence of PCa. The researchers attributed this high incidence to permissive opportunistic screening policies followed by primary care centres, involving serum PSA determinations in a population not recommended by clinical guidelines. Additionally, the study suggests that the same permissiveness extends to the departments involved in performing prostate biopsies in elderly patients, even in the absence of other poor parameters and despite a low probability of presenting a clinically significant tumour [28].

The study reporting about prostate cancer screening in the Czech Republic does not report details on the current situation of screening in the Czech Republic.

### 3.2. What Are the Policy Recommendations across the European Union for Future PSA Testing?

Based on the literature presented, future policy recommendations across the European Union regarding the early detection of prostate cancer can be inferred. The literature suggests several potential policy measures that could be considered across the EU or in respective countries.

Albreht et al. [15] suggested formulating and proposing revised guidelines on a comprehensive approach to control PCa, including screening across the European Union. This will provide a structured and organised approach to the early detection or risk-stratified screening for prostate cancer [15].

Kappen et al. [16] asked for a consistent approach towards PSA testing, especially among healthcare professionals in Germany and the Netherlands. In their research, they identified that in the Netherlands and Germany, urologists are more in favour of using PSA tests than GPs; however, instead of focusing on the different attitudes towards PSA testing, a consistent recommendation should be established [16].

Reljić et al. [24] highlighted the need for multidisciplinary discussion in Croatia. This can include initiating debates involving all relevant stakeholders on the benefits and harms of different screening programs. National organisations, professional societies, and committees should lead discussions to identify the best possible scenario in each country’s setting [24].

Conde et al. [26] stressed the need to implement global social marketing strategies to change the prevailing culture of excessive requests for laboratory tests in Portugal. This awareness-raising strategy may reduce overdiagnosis and overtreatment [26].

Westhoff et al. [23] emphasised the need for risk-adapted screening approaches based on various diagnostic options in addition to the PSA value in Germany. He argues that risk-adapted screening approaches should be more promoted to utilise the correct biopsy indication. These options may include identifying risk groups, baseline PSA, PSA density, PSA dynamics, multiparametric magnetic resonance image (MRI) of the prostate, and risk calculators [23].

Also, from a German perspective, Westhoff et al. [23] and Albreht et al. [15] stressed that policymakers and relevant stakeholders should consider the ongoing studies on risk-adapted strategies of PSA screening before making decisions on the introduction of population-based PSA screening. Evidence-based approaches should guide policy decisions [15,23].

## 4. Discussion

This systematic review highlights significant variations in the approach to PSA-based early detection for prostate cancer across different European countries. This is the first systematic review since 2016 which tried to map the current policies and state of play portrayed in the scientific literature across the European Union. We identified studies from nine European Union countries (Germany, France, the Netherlands, Croatia, Ireland, the Czech Republic, Lithuania, Portugal, and Spain). Only two countries (Germany and France) have a clear recommendation from the government against screening with respective guidelines to support the recommendations from health authorities [17,22,23]. Two articles about the Netherlands showed that there are guidelines in the Netherlands which recommend against PSA testing and, in addition, there seems to also be a recommendation against shared decision making regarding PSA testing [20,29].

Across all countries, with or without official recommendations from the medical authorities, there appears to be a high level of opportunistic screening. Little information is available on the use of MRI or biopsies. To reduce overdiagnosis and overtreatment, authors from different countries recommend various changes on a policy level. This includes clear guideline recommendations to enable a structured and organised approach to early detection or risk-stratified recommendations on early detection. Ultimately, increased guideline adherence by professional stakeholders should also include multidisciplinary discussions and social medical campaigns to raise awareness and an emphasis on evidence-based early detection approaches.

In their non-systematic review, Bratt et al. also examined current health policies and highlighted screening policies in countries like Lithuania and Sweden. In Lithuania, Bratt et al. discussed the opportunistic PSA screening program also identified in this review [30]. However, Lithuania is participating in the PRAISE-U project as a pilot site, aiming to align their current opportunistic screening approach with the risk-adapted algorithm proposed by PRAISE-U while formalising the invitation system [31]. In Sweden, the Swedish Ministry of Health and Social Affairs has assigned the Confederation of Regional Cancer Centres to standardise widespread prostate cancer testing and establish organised prostate cancer testing (OPT) programs. OPT sends invitations to men aged 50–74 years via a letter that neutrally informs them about the program. The OPT office manages all aspects, including testing intervals, the use of MRI, and potential biopsies, following an algorithm. Results are recorded for quality control and research purposes. OPT is regarded as a model for a successful smart early detection programme [30].

Another example of a country transitioning to an algorithm-based organised screening strategy is the Czech Republic. According to the national health statistics institute, currently, half of men aged 50 and older undergo unorganised PSA testing. This places a significant burden on the Czech healthcare system, with estimated costs of EUR 17 million for men aged 50–69 and nearly EUR 14 million for older men. These costs include testing and follow-up diagnostics. With the new guidance provided by the Council of the European Union, the Czech Republic has been working since 2022 to establish a new prostate cancer detection pilot program to be started from 2024. This effort included preparatory work such as policy roundtable discussions, an analysis of the current situation and the potential impact of an organised program, as well as the development of strategies and implementation guidelines for population pilot programs [32]. This aligns with the Council’s recommendation to consider piloting organised screening programs [12].

Contrary to their current recommendation, France has also introduced an opportunity to revise their stance on cancer screening in their updated 2021–2023 Ten-Year Cancer Control Strategy. They expressed their commitment to enhancing research in this area, with the goal of providing more effective screening programs and developing innovative screening methods for conditions like lung and PCa. Additionally, they aim to progress towards a more personalised screening approach that better considers the individual risk profile of each person [33].

Vickers et al. published a narrative review on current policies on early detection to highlight that testing purely based on shared decision making leads to inequitable screening favouring the wealthier and more educated men, as well as screening outside of the target age group [34]. They highlight policies which recommend an informed choice about testing from Australia, Canada, France, Germany, Ireland, Italy, Sweden, Switzerland, the United Kingdom, and the US. Instead of promoting informed choice solely on performing a PSA test or not, they recommend using a comprehensive, risk-based, prostate cancer detection programme [34]. This is in line with the policy recommendations presented in this systematic review. The high degree of evidence of opportunistic screening in countries like Germany and France, where there is currently a clear recommendation against PSA testing, shows that the current policy landscape seems to not be fit for purpose.

## 5. Conclusions

This systematic review sheds light on significant variations in the approach to PSA-based early detection for prostate cancer across different European countries, with high levels of opportunistic screening. The suggested policy recommendations aim to promote standardised guidelines, risk-adapted screening, informed decision-making, and increased awareness to enhance the effectiveness and efficiency of prostate cancer early detection in the European Union. Building on this evidence, PRAISE-U presents an opportunity to revolutionise prostate cancer screening methodologies by adopting a risk-based approach that aligns with European Union member states, as well as promoting positive change towards current awareness around PSA testing by incorporating the learnings of the last 30 years of research.

## Figures and Tables

**Table 1:** SPIDER inclusion and exclusion criteria.

Literature Search	
SPIDER (Sample, Phenomenon of Interest, Design, Evaluation, Research Type)	
Inclusion	Exclusion
Date: Published from 2016 onwards + (publication date)	Date: Published before 2016
Sample: Men screened in the European Union for prostate cancer; all stages of the screening process	Sample: European Union not mentioned; other cancer screening than prostate cancer
Phenomenon of Interest: Screening policies/recommendations or current practice (with focus on policy/recommendation) of European Union countries with a focus on PSA	Phenomenon of Interest: No policies, recommendations, or current practice of testing mentioned
Design: n/a	Design: n/a
Evaluation: Dependent on the type of study; not specific since the research type is inclusive.	Evaluation: n/a
Research type: Qualitative, quantitative, mixed methods, reviews	Research type: Abstracts

## Supplementary Materials

The following supporting information can be downloaded at: https://www.mdpi.com/article/10.3390/jpm14010084/s1, Supplementary Materials S1—Search Strategy.

## Disclaimer

Co-funded by the European Union. Views and opinions expressed are, however, those of the author(s) only and do not necessarily reflect those of the European Union or HaDEA. Neither the European Union nor the granting authority can be held responsible for them. Where authors are identified as personnel of the International Agency for Research on Cancer/World Health Organization, the authors alone are responsible for the views expressed in this article, and they do not necessarily represent the decisions, policy, or views of the International Agency for Research on Cancer/World Health Organization.

## Appendix A. The PRAISE-U Consortium

Hendrik Van Poppel (EAU), Sarah Collen (EAU), James N’Dow (EAU), Phillip Cornford (EAU), Juan Gómez Rivas (EAU), Monique Roobol-Bouts (EMC, ERSPCF), Renée Leenen (EMC), Lionne Venderbos (EMC, ERSPCF), Jozien Helleman (EMC), Renée Leenen (EMC), Daan Nieboer (EMC), Pia Kirkegaard (CDR), Berit Andersen (CDR), Louise Dybdahl Pedersen (CDR), Mette Bach Larsen (CDR), Sofie Meyer Andersen (CDR), Grace McKinney (CDR), Karel Hejduk (UZIS), Ondřej Májek (UZIS), Ondřej Ngo (UZIS), Tomáš Vyskot (UZIS), Marcela Koudelková (UZIS), Roman Zachoval (UZIS, CUS), Renata Chloupkova (UZIS), Katerina Hejcmanova (UZIS), Roderick van den Bergh (UMCU), Peter-Paul Willemse (UMCU), Norbert Couespel (ECO), Riccardo Moschetti (ECO), Nora Lorenzo (ECO), Mike Morrissey (ECO), Richard Price (ECO), Enea Venegoni (ECO), Agnese Konusevska (ECO), Ana Mula (ECO), Dorota Dudek-Godeau (DCOPiH, NIZP), Malgorzata Krynicka (DCOPiH), Krzysztof Tupikowski (DCOPiH), Kataryzyna Hodyra-Stefaniak (DCOPiH), Monika Pajewska (NIZP), Aleksandra Czerw (NIZP), Andrzej Deptała (NIZP), Ángel Gómez Amorín (CSG), Silvia Suárez Luque (CSG), Carmen Durán Parrondo (CSG), Ana Marina Tarrazo Antelo (CSG), Josep Vilaseca (ALT, WONCA), Gemma Cuberas (ALT), Anna Arnau Bartés (ALT), Juan Pablo Salazar (ALT), Hector Lopez Llaurando (ALT), Ola Bratt (VGR), Rebecka Godtman (VGR), Emil Järbur (VGR), Thomas Jiborn (SKA), Anders Bjartell (SKA), Anna Holst (SKA), Max Alterbeck (SKA), Aušvydas Patašius (NCI), Gintare Miksiene (NCI), Adomas Ladukas (NCI), Giedrė Smailytė (NCI), Ernestas Janulionis (NCI), Zygimantas Kardelis (NCI), Marius Kincius (NCI), Mingaile Drevinskaite (NCI), Auguste Kaceniene (NCI), Lieven Annemans (UG), Pieter-Jan Hutsebaut (UG), Pieter Vynckier (UG), Robert Kidd (HSE), Michael O Brien (HSE), Paula Keon (HSE), Carolyne Lynch (HSE), Michael Rooney (HSE), Martin Kivi (EUS), David Galvin (UCD), Eamonn Rogers (UCD), Eileen Nolan (UCD), Paul Seeney (UCD), Alexander Bauer (WONCA), Thomas Frese (WONCA), Christine Bruetting (WONCA), Cate Bennett (MOV), Amy O’Connor (MOV), Sarah Coghlan (MOV), Ricky Le Roux (MOV), Karen Robb (MOV), Partha Basu (IARC), Arunah Chandran (IARC), Andre Carvalho (IARC), Deependra Singh (IARC), Lobna Boulegroun (IARC), Milagros Otero-García (ESUR), Erik Briers (Europa UOMO), Anna Lantz (RS), Lisa Jelf Eneqvist (RS)

STICHTING EUROPEAN UROLOGICAL FOUNDATION (EAU), established in Mr. E.N. van Kleffensstraat 5, 6842 CV Arnhem, Netherlands;

ERASMUS UNIVERSITAIR MEDISCH CENTRUM ROTTERDAM (EMC), established in Dr Molewaterplein 40, Rotterdam 3015 GD, Netherlands;

STICHTING EUROPESE STUDIE PROSTAATKANKER SCREENING (ERSPCF), established in Schaarweide 5, Reeuwijk 2811 JM, Netherlands;

REGION MIDTJYLLAND (CDR), established in Skottenborg 26, Viborg 8800, Denmark;

USTAV ZDRAVOTNICKYCH INFORMACI A STATISTIKY CESKE REPUBLIKY (UZIS), established in Palackeho Namesti 4, Praha 12801, Czechia;

UNIVERSITAIR MEDISCH CENTRUM UTRECHT (UMCU), established in Heidelberglaan 100, Utrecht 3584 CX, Netherlands;

EUROPEAN CANCER ORGANISATION (ECO), established in Rue De La Science 41, Bruxelles 1040, Belgium;

DOLNOSLASKIE CENTRUM ONKOLOGII, PULMONOLOGII I HEMATOLOGII (DCOPiH), established in Ul. Pl. Ludwika Hirszfelda, 12 000, 53-413, Wroclaw, Poland;

NARODOWY INSTYTUT ZDROWIA PUBLICZNEGO PZH—PANSTWOWY INSTYTUT BADAWCZY (NIZP), established in Chocimska 24, Warszawa 00791, Poland;

CONSELLERIA DE SANIDADE DE GALICIA (CSG), established in Edificio Administravito San Lazaro sn, Santiago de Compostela 15781, Spain;

ALTHAIA XARXA ASSISTENCIEL UNIVERSITARIA DE MANRESA FUNDACIO PRIVADA (ALT), established in Calle Doctor Joan Soler 1–3, Manresa, Barcelona 08243, Spain;

VASTRA GOTALANDSREGIONEN (VGR), established in Regionens Hus, Vanersborg 462 80, Sweden;

REGION SKANE (SKA), established in Region Skane, Kristianstad 291 89, Sweden;

NACIONALINIS VEZIO INSTITUTAS (NCI), established in Santariskiu Str, 1, Vilnius 08660, Lithuania;

GHENT UNIVERSITY (UG), public institution with legal personality, having its administrative offices at Sint-Pietersnieuwstraat 25, B-9000 Gent, Belgium;

HEALTH SERVICE EXECUTIVE (HSE), established in Limetree Avenue, Millenium Park, NAAS Nass, County Kildare, Ireland;

EESTI UROLOOGIDE SELTS (EUS), established in L Puusepa TN 8, Tartu 51014, Estonia;

UNIVERSITY COLLEGE DUBLIN, NATIONAL UNIVERSITY OF IRELAND, DUBLIN (UCD), established in Belfield, Dublin 4, Ireland;

STICHTING WONCA EUROPE (WONCA), established in Poljanski Nasip 58, Ljubljana 1000, Slovenia;

MOVEMBER FOUNDATION EV (MOV), established in Leopoldstr 11 A, Munchen 80802, Germany;

INTERNATIONAL AGENCY FOR RESEARCH ON CANCER (IARC), the cancer research agency of the World Health Organization (WHO), whose offices are at 25 avenue Tony Garnier, CS 90627, 69366 LYON CEDEX 07, France

EUROPEAN SOCIETY OF UROGENITAL RADIOLOGY (ESUR), whose administrative offices are at Landstrasser Hauptstrasse 27, Eingang Weyrgasse 9 / Tür 15, 1030, Vienna, Austria;

EUROPA UOMA (Europa UOMO), whose administrative offices are atLeopoldstraat 34 000, 2000, Antwerpen, Belgium;

THE CZECH UROLOGICAL SOCIETY (EUS), whose administrative offices are at Sokolská 490/31, 120 00, Praha 2, Czechia;

REGION STOCKHOLM (RS), whose administrative offices are at Hantverkargatan 45 22550, 104 22, Stockholm, Sweden;
