# Supplementary material for: Health Policy for Prostate Cancer Early Detection in the European Union and the Impact of Opportunistic Screening: PRAISE-U Consortium
Source: J Pers Med. 2024 Jan 11;14(1):84. doi: 10.3390/jpm14010084 (PMC10819943; doi:10.3390/jpm14010084)
Supplement: Supplementary file 1 [file jpm-14-00084-s001.zip › jpm-2770416-supplementary.pdf]

Supplementary Materials S1- Search Strategy

prostate cancer screening policy

grey literature

google scholar

google

policy commons

embase

('prostate tumor'/exp OR 'prostate specific antigen'/de OR 'digital rectal examination'/de OR 'prostate biopsy'/de OR (prostate/exp AND ('nuclear magnetic resonance imaging'/exp OR echography/exp)) OR ((prostat* NEAR/6 (tumor* OR tumour* OR cancer* OR neoplas* OR antigen* OR ultraso* OR mri OR magnetic-resonan* OR biops*)) OR psa OR (digital* NEAR/3 rectal* NEAR/3 examinat*) OR proPSA OR fPSA OR Prostate-Health-Index):ab,ti,kw)

AND

(screening/de OR 'mass screening'/de OR 'screening test'/de OR rescreening/de OR 'cancer screening'/de OR 'early cancer diagnosis'/de OR 'early diagnosis'/de OR (screening OR rescreening OR (early NEAR/3 (deect* OR diagnosis))):ab,ti,kw)

AND

(policy/exp OR politics/de OR Government/de OR 'political activism'/de OR lobbying/exp OR 'health insurance'/de OR reimbursement/de OR 'biomedical technology assessment'/de OR (policy OR policies OR politic* OR Government* OR lobby* OR insurance* OR reimburse* OR re-imburse* OR biomedical-technology-assessment* OR health-technology-assessment*):ab,ti,kw)

AND

('European Union'/de OR 'EU citizen'/exp OR Austria/de OR Belgium/de OR Bulgaria/de OR Croatia/de OR Cyprus/de OR 'Czech republic'/de OR Denmark/de OR 'The netherlands'/de OR Estonia/de OR Finland/de OR France/de OR Germany/de OR Greece/de OR Hungary/de OR Ireland/de OR Italy/de OR Latvia/de OR Lithuania/de OR Malta/de OR Poland/de OR Portugal/de OR Romania/de OR Slovakia/de OR Slovenia/de OR Spain/de OR Sweden/de OR (European-Union OR Austrian* OR Belgian* OR Bulgarian* OR Croatian* OR Cypriot* OR Czech* OR Danish* OR Dutch* OR Estonian* OR Finn* OR French* OR German* OR Greek* OR Hungarian* OR Irish* OR Italian* OR Latvian* OR Lithuanian* OR Maltese* OR Polish* OR Portuguese* OR Romanian* OR Slovak* OR Slovenian* OR Spaniard* OR Swedish* OR Austria* OR Belgium* OR Bulgaria* OR Croatia* OR Cyprus* OR Denmark* OR netherland* OR Estonia* OR Finland* OR France* OR Germany* OR Greece* OR Hungary* OR Ireland* OR Italy* OR Latvia* OR Lithuania* OR Malta* OR Poland* OR Portugal* OR Romania* OR Slovakia* OR Slovenia* OR Spain* OR Sweden*):ab,ti,kw)

Mesh: Policy Making
